# Analysis of LruC lipoprotein and identification of peptides candidates for vaccine development and diagnosis of leptospirosis

**DOI:** 10.1371/journal.pone.0281344

**Published:** 2023-02-06

**Authors:** Iago H. de Miranda Mariano, Bruna Ferreira Silva, Mayriele da S. Machado, Roberta M. Blanco, Eliete C. Romero, Sonia A. Andrade, Paulo Lee Ho, Elizabeth A. L. Martins, Josefa Bezerra da Silva

**Affiliations:** 1 Laboratory of Bacteriology, Butantan Institute, Sao Paulo, Brazil; 2 PIBITI/CNPq and Butantan Foundation, São Paulo, Brazil; 3 Laboratory of Bacteriology, Adolfo Lutz Institute, Sao Paulo, Brazil; 4 Laboratory of Biopharmaceuticals, Butantan Institute, Sao Paulo, Brazil; 5 Bioindustrial Division, Butantan Institute, São Paulo, Brazil; 6 Laboratory of Recombinant Biological, Butantan Institute, São Paulo, Brazil; Cornell University, UNITED STATES

## Abstract

Leptospirosis is a public health concern with lethality around 15% of the total cases. The current vaccines against *Leptospira* infection based on bacterins have several limitations, which require urgent development of new ones. In this context, groundbreaking approaches such as peptide-vaccines could be used to come around with promising results. Our goal was to identify conserved and immunogenic epitopes from the lipoprotein LruC that could interact with Major Histocompatibility Complex (MHC) I and II. LruC is a conserved lipoprotein expressed during leptospirosis that is considered among vaccine candidates and can be used as source for development of peptide-based vaccines. We searched for peptides that would be recognized by antibodies from either serum of hamsters previously immunized with low-LPS bacterin vaccines or from serum of patients diagnosed with leptospirosis. Immuno properties of seven peptides from LruC protein were evaluated *in silico* and by Dot Blot assay, and validate by ELISA. Preliminary results pointed one promising peptide that was recognized by the sera. In conclusion, the immunoinformatic approach helps the search and screening of peptides, while the Dot Blot assay, a simple and effective tool, helps to test and validate them. Thus, these prospective techniques together were validated to identify and validate potential peptides for further investigation as peptide-based vaccines or diagnostic methods.

## Introduction

Leptospirosis is a zoonotic widespread disease caused by pathogenic bacteria species of the genus *Leptospira*. The disease is caused by direct contact with infected animals or by indirect contact via contaminated environment, where the main hosts are rodents, dogs, cattle, and humans [1, 2]. There are about one million human cases and 60,000 deaths per year worldwide, with a growing number of countries reporting leptospirosis outbreaks [3, 4].

Vaccination is the most effective strategy to decrease the impact on public health, preventing the disease [5]. The current anti-leptospirosis vaccines are based on inactivated whole-cell preparations, bacterins, but they have several limitations, such as side effects, short-term immunity, serovar-restricted protection [6] and the need of annual vaccinations due to T-independent nature of the response elicited by LPS [7]. As a result, only a few countries with high-risk populations have used bacterins to control leptospirosis [7]. Consequently, researches have been looking for alternative technologies for vaccine development. Peptide-based vaccines are considered a promising strategy against pathogenic *Leptospira*, designed to contain immunogenic and conserved epitopes able to induce broad-spectrum immunity against multiple pathogenic *Leptospira* serovars. These epitopes should be antigenic fragments of larger proteins and able to induce cellular and humoral responses with cross-protection capacity [1, 2, 6, 8]. Different bacterial outer membrane proteins (OMPs) were tested as candidates for immunization due to their abundance and accessibility, such as LipL32, OmpL1, and LipL41. However, they all failed to provide cross-protection and showed low efficacy until now [9–11]. Other *Leptospira* proteins, LigA and LigB, two conserved outer membrane proteins with immunoglobulin-like motifs, have been tested as vaccines in many strategies. Although LigA demonstrated significant protection, it did not show heterologous protective effect [6].

The advances in bioinformatics has emerged with helpful tools in the identification of epitope candidates for vaccines and diagnostics development [12–14]. Immunoinformatics is currently based on genome and proteome information to predict B-cell and T-cell epitopes with a higher potential to be recognized by the immune system, accelerating the process of search and possibly avoiding large number of *in vivo* investigation [6, 15]. Immunoinformatics analysis suggested LruC protein, which is a conserved lipoprotein present in the inner portion of the *Leptospira* outer membrane, as an optimistic vaccine candidate. Several epitopes of different proteins including LruC, were suggested as interesting for peptide-based vaccines studies due to their conservation and potential to interact with MHC I and II molecules [16, 17].

Therefore, our goal was to analyze LruC peptides that could be recognized by antibodies of either serum from hamsters immunized with low LPS bacterin or serum from patients diagnosed with leptospirosis, thus selecting potential epitopes to be used in the development of peptide-based vaccines.

## Materials and methods

### Peptides selection and in silico analyze

The peptides were first selected by Lata et al., (2018) [16], using Immune Epitope Database (IEDB) to predict linear B-cell epitopes, NetCTL 1.2 server to predict for T-cell epitopes, and a series of other bioinformatic tools to analyze the features for final selection of the peptides. We analyzed six LruC derived peptides by Uniprot bioinformatics tool Basic Local Alignment Search Tool (BLAST protein) to confirm their protein source and the conservation among *Leptospira* species. Seven selected peptides sequences were analyzed with NetMHC 4.0 (https://services.healthtech.dtu.dk/service.php?NetMHC-4.0) and NetMHCIIpan 4.0 (https://services.healthtech.dtu.dk/service.php?NetMHCIIpan-4.0) against 12 and 24 MHC alleles respectively in order to evaluate their binding capacity to MHC molecules I and II. The full LruC sequence was analyzed by the programs and several overlapping peptides were indicated as recognized by MHC complex, around the core we called Pep 7, pointed as having "strong biding" capacity.

### Peptides synthesis and purification

The peptides were analyzed and Pep-1 and Pep-2 had one amino acid modified (indicated in bold) to facilitate the synthesis. The sequences were Pep1-GSIPFTYNTV**G**QT, Pep2-V**G**TIPLNLVVTD, Pep3-AEGVSTVAYEDLYPSA, Pep4-YSSSFILIIKKG, Pep5-TKTVSSSD, Pep6-LGSYPYDIFIKVK, Pep7-WAILVPGA. All seven peptides were synthesized and purified by High Performance Liquid Chromatography (HPLC) or bought from Proteimax company. Peptides were freeze-dried and stored at 2-8° C.

### Preparation of *Leptospira* extracts for antibody recognition analysis

*Leptospira* extracts were prepared as described previously [18] from *L*. *interrogans* serovar Copenhageni and *L*. *biflexa* serovar Patoc to be used as positive and negative control respectively. Briefly, the strains were cultured in EMJH liquid medium for 6 days, centrifuged, and cellular pellets washed with PBS. Next, the bacteria were suspended in PBS with protease inhibitor cocktail (Sigma), and then lysed by glass beads in a Beads beater (Biospect Products), 2500 rpm for 2min. The tubes were immersed immediately in ice and the process was repeated 10 times. Total protein was quantified in Nanodrop 1000, aliquoted and storage at -80°C. *Escherichia coli* LPS (Sigma, US) was used as a negative control.

### Primary antibody from hamsters

Sera from hamster immunized with low LPS vaccines were obtained by Lauretti et al. (2020) [18]. Survivor immunized animals were euthanized 30 days after the challenge with virulent *L interrogans*, and the serum was collected according to protocol described and approved by CEAUIB n°. 1314/14 and n°. 1255/14. The animals were obtained from animal house of Instituto Butantan or Instituto de Ciências Biomédicas da Universidade de São Paulo. Animals were manipulated and daily monitored by trained personnel. The approved protocols describe the method of euthanasia of the animals by controlled flow rate of CO_2_ in a closed chamber. After euthanize, approximately 1 mL of blood was recovered by heart puncture from hamster and or 0.2 mL from mice. Lung, liver and kidney were collected in plastic tube and immediately submerse into liquid nitrogen, and kept to -80°C for RNA purification and further analysis.

### Primary antibody from patients diagnosed with leptospirosis

Samples of serum from individuals diagnosed with leptospirosis were obtained from Adolfo Lutz Institute, Sao Paulo/SP, institution responsible for the diagnosis of leptospirosis. The need for consents from the donors of samples was waived by the National Research Ethics Committee (CONEP—Comissão Nacional de Ética em Pesquisa), according to the approved protocol n° 31938820.3.1001.0059. Serum samples were stored (-80°C) at Adolfo Lutz Institute after performing Micro agglutination test (MAT) for the routine leptospirosis diagnosis.

### Dot blot assay using synthetic peptides based on LruC protein

Five milligrams of freeze-dried peptides identified as Pep1 to Pep7 were diluted in ultrapure water at a final concentration of 50 μg/μL and 5 μL was applied on the surface of 0.1 or 0.45 μm Nitrocellulose membranes 2 x 6cm (Amersham Protran Premium, Germany). The membranes were dried for 30 min at room temperature and 30 min at 37°C before blocking 1 hour with 5 mL of 10% non-fat dry milk in 0.05% Tween-20 in PBS (TPBS). After blocking, membranes were washed with TPBS three times for 10min under agitation. The primary antibody was diluted depending on the assay. For sera from hamsters, the primary antibody consisted of pools of four or five samples of serum from the immunized or non-immunized animals, diluted 1:2000, samples of sera from leptospirosis patients or control were diluted according to MAT titers, ranging from 1:500 to 1:2000 in 5% non-fat dry milk in 0.05% TPBS. The membranes were incubated with the correspondent serum for 2 h. Next, the membranes were washed with TPBS again and incubated with the secondary antibody, rabbit anti-hamster or goat anti-human conjugated with peroxidase (Invitrogen, USA) in TPBS 1:3000 for 1 h. The last wash was performed within 0.05% Tween-20 in TBS (150mM NaCl, 24 mM Tris base, 2.7 mM KCl) and membranes were then developed using ECL reagents 1:1.

The chemiluminescent signals were detected at Cambridge Uvitec and images were analyzed using ImageJ software to calculates the density of the spots. The background of the membrane was subtracted from the intensity of the experimental spots and the results present the difference of intensity of the spots developed by the positive sera samples and the spots using control samples.

### Validation of peptides recognition by anti-Leptospira human antibodies, by ELISA

The ELISA was performed according Silva and collaborators (2020) [19], with some modifications. Briefly, 96 well high biding plates were coated in duplicate with 2.5, 10 or 25 μg of peptides diluted in sodium carbonate/bicarbonate buffer pH 9.5 and incubated at 4°C, overnight. Then plates were washed and blocked with 10% nonfat dried milk in PBST solution (PBS with 0.05% Tween 20) for 1h at 22°C. Plates were then washed 3 times with PBST and incubated for 2 h with 100 μL of human sera (1:500) diluted in PBST with 5% nonfat dried milk. After washing with PBST, plates were incubated for 2 h with goat anti-human antibody conjugated with peroxidase (Invitrogen^®^ - 1:5000 diluted in PBST). Then 100 μL of R&D systems^®^ reagents, solution mix 1:1 A (H2O2) and B (tetramethylbenzidine), were applied and the plates were incubated for 25 min. The reaction was stopped with 50 μL of stop solution (2 N H2SO4) and the optical density determined at 450 nm.

### RNA extraction and quantification of LruC gene expression by PCR (qPCR)

LruC gene expression was determined in mice organs samples by qPCR after extraction of RNA and synthesis to cDNA [19]. Total RNA was extracted using RNeasy mine kit (Qiagen) according to the manufacturer’s instructions and quantified in a NanoDrop 1000 spectrophotometer. Quantitative PCR was performed in a total reaction volume of 12.0 μL, containing 0.4 μL of each LruC or 16S primer, 6.25 μL real-time PCR SYBR green Master Mix (Applied Biosystems), 1 μL of cDNA template of 1:10 dilutions and sterilized deionized water up to the final reaction volume. All qPCRs reactions were performed in triplicate using the primers forward-ACGCACAAACCGGCTATAA and reverse-CGGGAATACCTTTGCTTGATTG for LruC gene, and the primeres forward-TTCAGTTGGGCACTCGTAAG and reverse-CGTGTGTTGCCCTAGACATAA for *Leptospira* gene 16S as endogenous normalized. The qPCR assay was performed and analyzed using Applied Biosystems 7300 Real-Time PCR system and its efficiency was determined for each reaction using software LinRegPcr. All oligonucleotides had the correlation coefficient squared (R2) higher or equal to 0.998 and their efficiency range was in between 1.9–2.0, which indicates stable and reliable assay.

### Statistic analysis

Statistical analysis to compare LruC gene expression during infection were evaluated by two-way ANOVA alpha. Statistical analysis and plotting of data were performed using Prism software (GraphPad).

## Results

### In silico analysis of selected peptides

**Fig 1:**
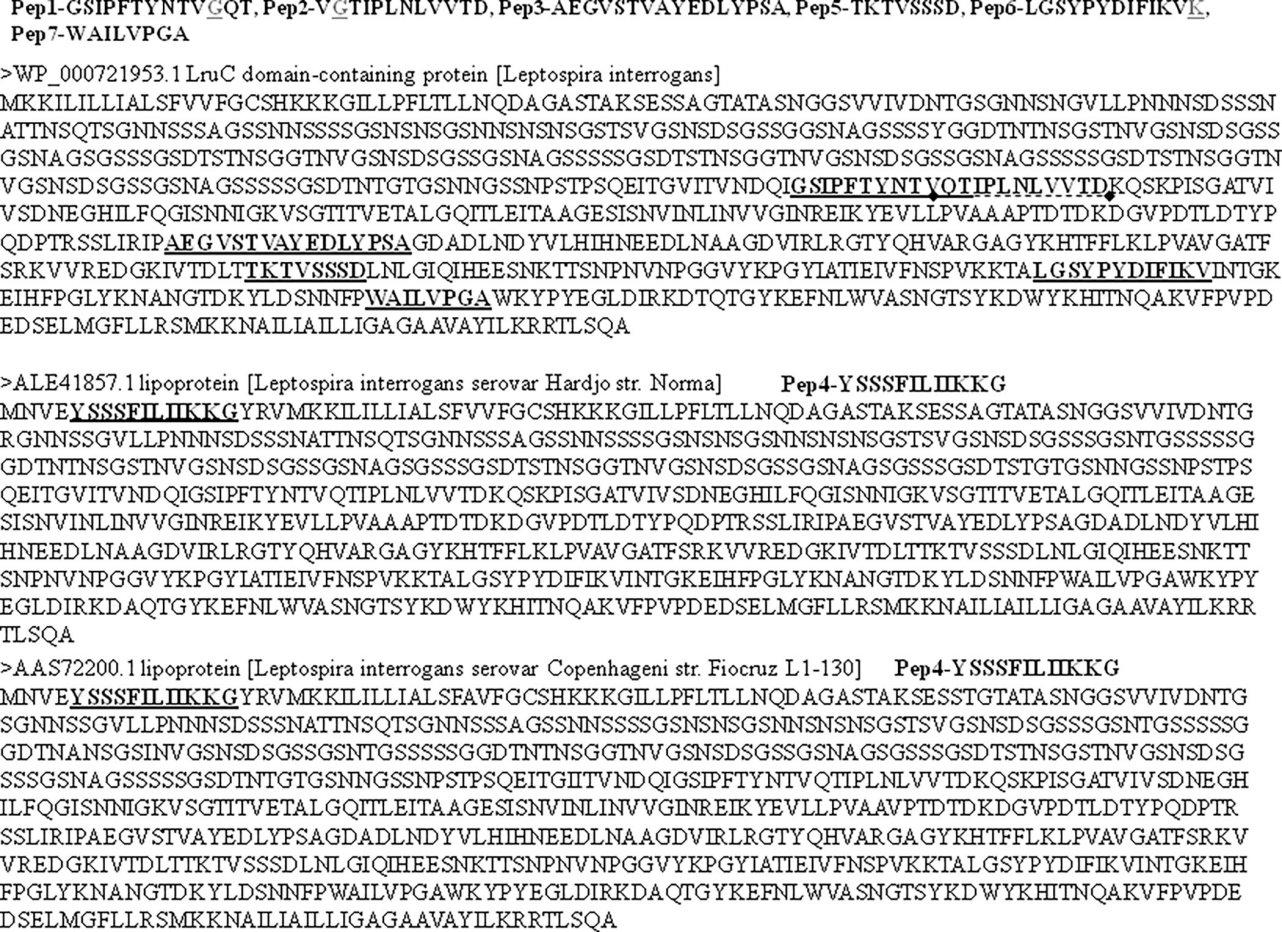
*Leptospira* proteins showing the peptides sequences.

The protein source of the peptides was confirmed by BLAST protein of Uniprot (Fig 1). Six of the seven peptides matched to *Leptospira* LruC protein (WP_000721953.1), and they were conserved among several *Leptospira* pathogenic species (S1 Table in S1 File). The Pep4 showed similarity with other *Leptospira* proteins (S1 Table in S1 File).

### Peptides synthesis and purification

**Fig 2:**
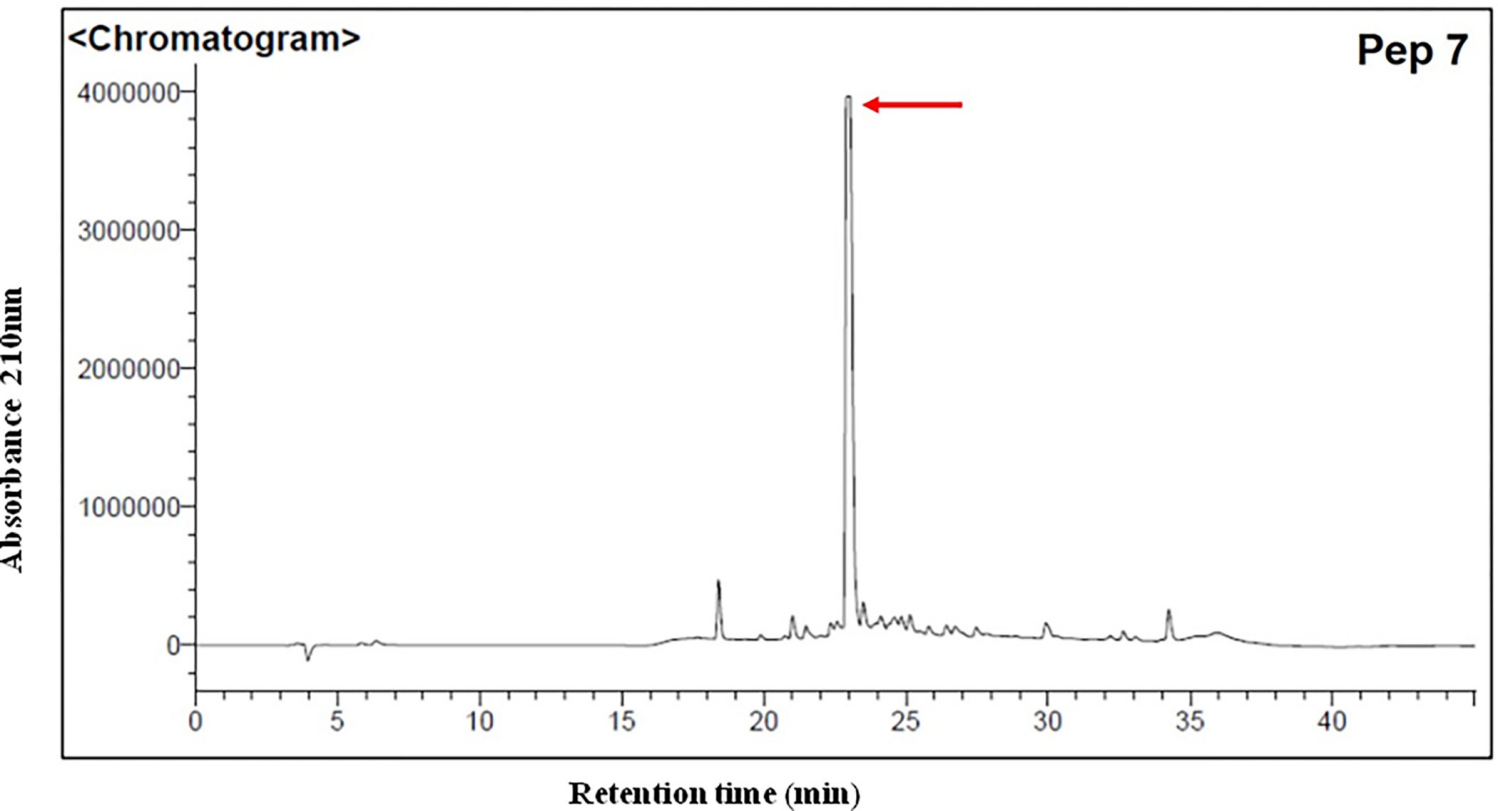
HPLC chromatogram representing the peak of peptide 7 after purification as example.

The selected peptides were chemically synthesized and purified by HPLC. All peptides, except the Pep4, presented defined peaks indicating a good purification as exemplified (Fig 2 and S1 Fig). Pep5 exhibited a well-defined peak, but low absorbance when compared to the other ones (S1 Fig).

### Identification of B-cell and T-cell epitopes

The peptides published by Lata and collaborators [16] were analyzed in terms of their capacity to interact with MHC II and I, using NetMHCpan II 4.0 and NetMHC 4.0 respectively (S3 Table in S1 File). Both tools are able to predict the binding of peptides to some HLA alleles using artificial neural networks (ANNs) [20, 21]. The peptides identified with higher potential to interact with MHC molecules would be better recognized by the immune system.

LruC protein sequence (WP_000721953) was analyzed using these tools and the resulting peptides were compared to the ones detected by Lata and collaborators [16]. Four of the seven peptides matched with MHC I and II with one or two extra amino acid (represented at Table 1 and S1-S3 Tables in S1 File in bold and underline), when compared to results from Lata and collaborators [16] (Table 1 and S1-S3 Tables in S1 File).

**Table 1:** Peptide 7 binding capacities against MHC I and II alleles.

Pep	HLA	Peptide	1-log50k(aff)	Affinity(nM)	%Rank	MHC II
**Pep7**	HLA-A0101	**P**WAILVPGA	0.061	25872.62	21.00	DRB1_0101
		WAILVPGA**W**	0.082	20493.68	10.00	DRB1_0102
	HLA-A0201	**P**WAILVPGA	0.049	29551.94	46.00	DRB1_0103
		WAILVPGA**W**	0.089	18992.46	26.00	DRB1_0301
	HLA-A0301	**P**WAILVPGA	0.022	39319.31	75.00	DRB1_0305
		WAILVPGA**W**	0.045	30564.25	40.00	DRB1_0401
	HLA-A2402	**P**WAILVPGA	0.059	26373.16	19.00	DRB1_0402
		WAILVPGA**W**	0.065	24688.98	17.00	DRB1_0403
	HLA-A2601	**P**WAILVPGA	0.037	33516.86	50.00	DRB1_0404
		WAILVPGA**W**	0.119	13812.78	5.00	DRB1_0405
	HLA-B0702	**P**WAILVPGA	0.036	33991.99	47.00	DRB1_0408
		WAILVPGA**W**	0.136	11515.49	8.00	DRB1_0701
	HLA-B0801	**P**WAILVPGA	0.023	38786.92	90.00	DRB1_0801
		WAILVPGA**W**	0.099	17164.90	19.00	DRB1_0803
	HLA-B2705	**P**WAILVPGA	0.047	30183.05	40.00	DRB1_0901
		WAILVPGA**W**	0.063	25317.94	28.00	DRB1_1001
	HLA-B3901	**P**WAILVPGA	0.021	39938.88	70.00	DRB1_1101
		WAILVPGA**W**	0.149	10005.26	4.50	DRB1_1104
	HLA-B4001	**P**WAILVPGA	0.035	34401.57	60.00	DRB1_1201
		WAILVPGA**W**	0.082	20486.58	13.00	DRB1_1301
	HLA-B5801	**P**WAILVPGA	0.039	32922.73	70.00	DRB1_1501
		WAILVPGA**W**	0.758	13.70	0.08 (SB)a	DRB3_0101
	HLA-B1501	**P**WAILVPGA	0.039	32806.09	60.00	DRB3_0202
		WAILVPGA**W**	0.183	6907.73	11.00	DRB4_0101

^a^Strong Binding.

Peptide 7 showed higher potential to be recognized by MHC I and II alleles, indicated as having strong biding to HLA. Besides, matched to different alleles’ representatives of HLA-A and HLA-B super types (Table 1 and S3 Table in S1 File).

For this analysis Pep3 with 16-mer, had to be divided in two parts and both were pointed out to be able to interact with MHC complex without any amino acid extension as compared to sequences detected by Lata et al (2018) [16]. Both parts of Pep3 seemed to interact also with HLA-A and HLA-B super type as well as MHC II (S3 Table in S1 File).

Differently, Pep1 sequence was shown to bind with HLA-A and HLA-B super types in MHC I complex with only one amino acid extension, but there were no correspondents in the MHC II analysis.

Finally, Pep2 and Pep4 had no correspondents at all with the immune complexes, suggesting that they would not interact with MHC complex, and consequently, not being able to stimulate the adaptive immune response.

### Peptides recognition by anti-leptospira antibodies from immunized hamster

**Fig 3:**
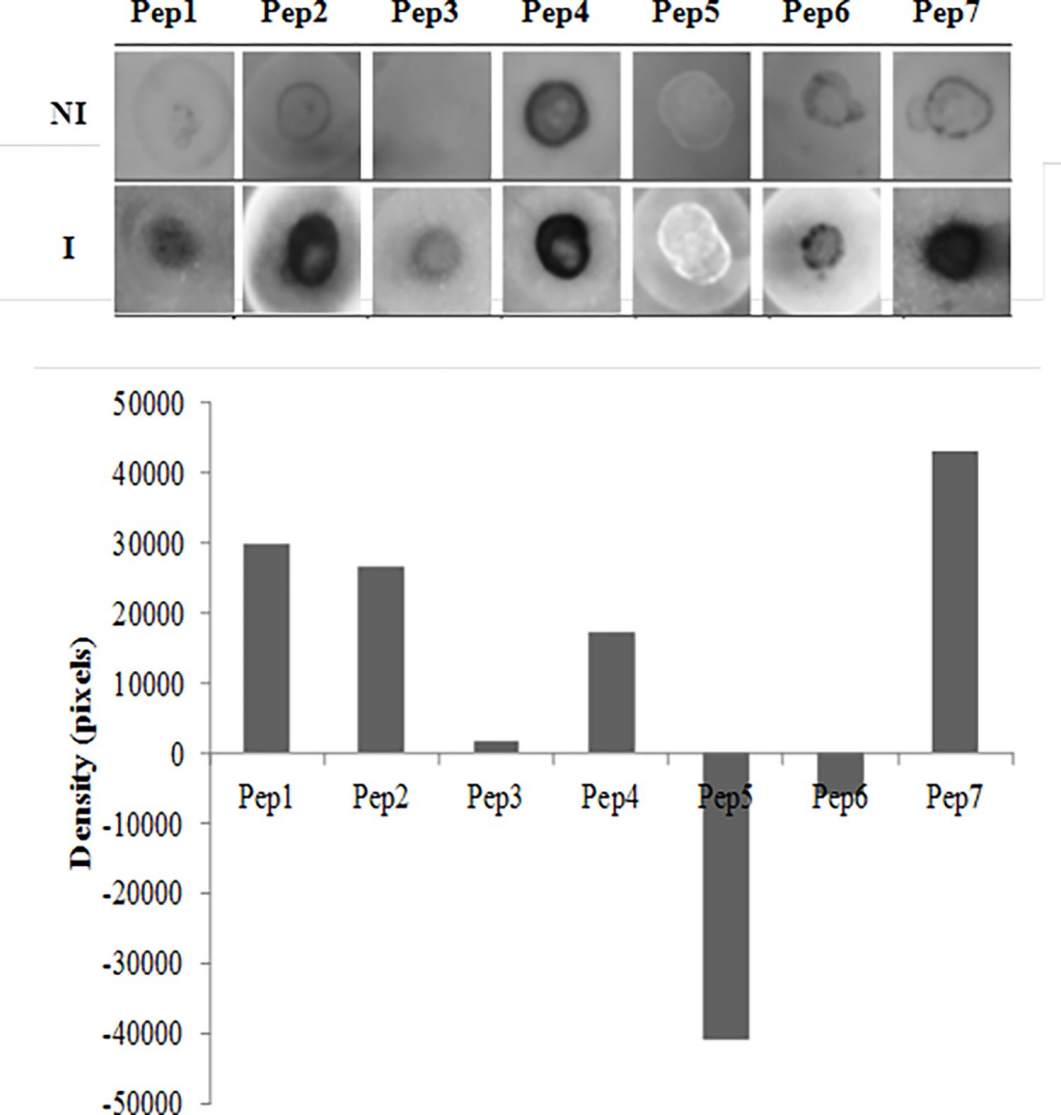
Recognition of peptides by antibodies in the sera of immunized hamsters. a) Dot Blot assay were set up with peptides bound to the membranes for interaction with sera from hamsters immunized with inactivated low-LPS vaccine (I) or non-immunized (NI). b) Quantification of the intensity of the spots by Image J software.

To investigate possible reactivity of the seven selected peptides with antibodies from previously immunized hamsters, we performed the dot blot assay. The Pep1, Pep2, Pep4, and Pep7 showed high chemiluminescent signals resulting from their interaction with the antibodies in hamsters’ sera (Fig 3 and S2 Fig), Pep1, Pep2, and Pep7 exhibited higher specificity, considering the recognition by control serum, non immunized hamsters. Differently, Pep4 exhibited low specificity, being recognized by the antibodies from control sera. Pep7 showed the highest chemiluminescent signal, while Pep3, Pep5, and Pep6 did not show significant interaction with antibodies from immunized or control group of animals (Fig 3).

### Peptides recognition by antibodies from sera of human diagnosed with leptospirosis

**Fig 4:**
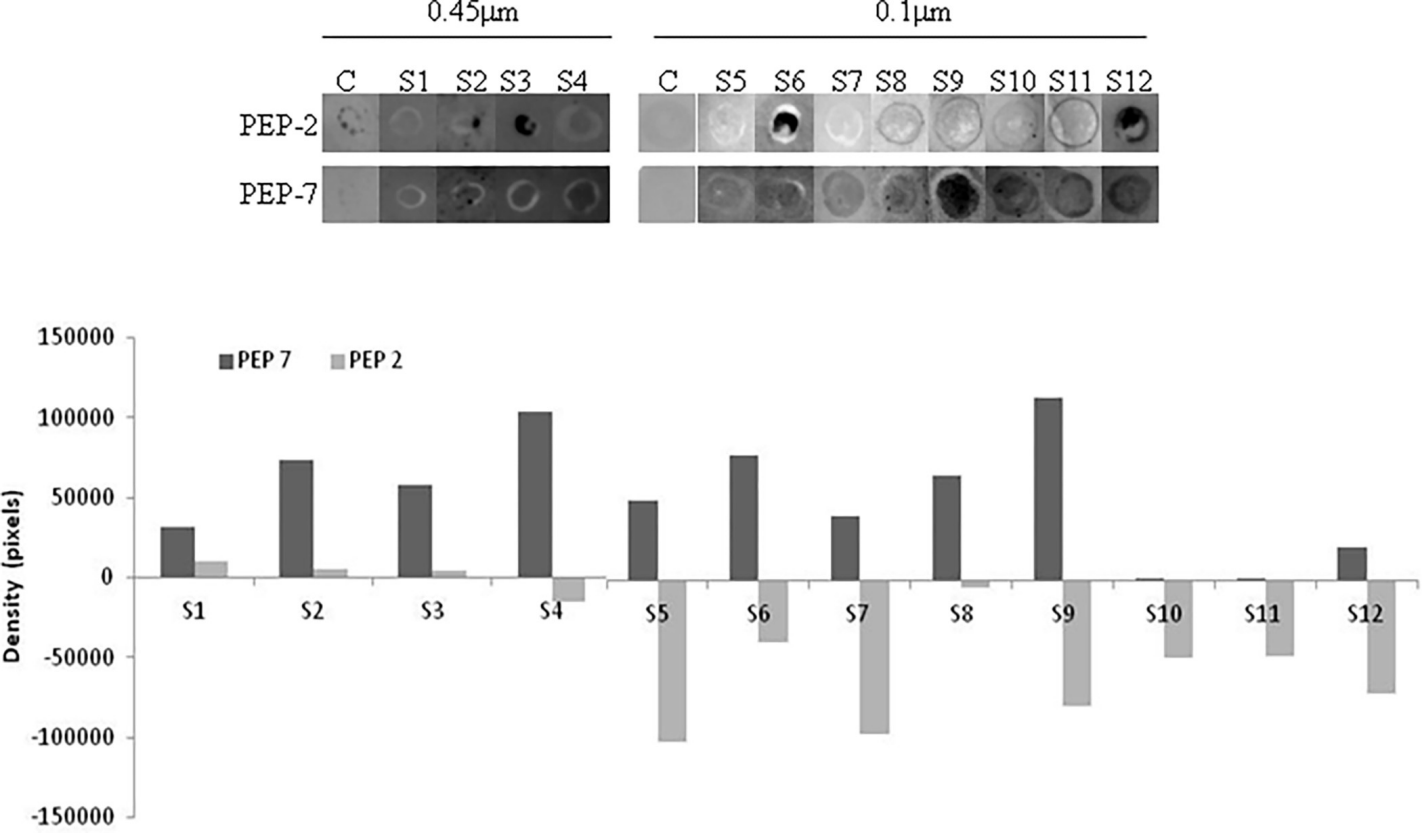
Recognition of Pep2 and Pep7 of LruC protein by sera from leptospirosis patients. Nitrocellulose membranes (0.45 or 0.1μm) of Dot Blot assay exhibiting the interaction of the selected peptides and the sera of leptospirosis patients (S1 to 12) and control (C), is shown in the top figure. The graphic represents the density of chemiluminescent signals resulting of Pep2 and Pep7 incubated with serum from different leptospirosis patients quantified using ImageJ software.

According to previous results, Pep2 and Pep7 were selected for analysis with sera from leptospirosis patients. Higher specificity was detected with Pep7 that was recognized by all leptospirosis positive sera and not by control sera (Fig 4).

Different levels of chemiluminescence were detected in the membranes incubated with the sera from leptospirosis patients. Highest signals were detected using Pep7 and samples of sera S4 and S9, and lowest signals with samples S1, S10, S11 and S12, when compared to the control sera from individual leptospirosis negative (C).

Pep2 showed lowest signals with sera from leptospirosis positive patients, which implies a lack of specificity of this peptide. These results reinforce our previous analysis indicating that Pep2 had no correspondent in MHC alleles.

**Fig 5:**
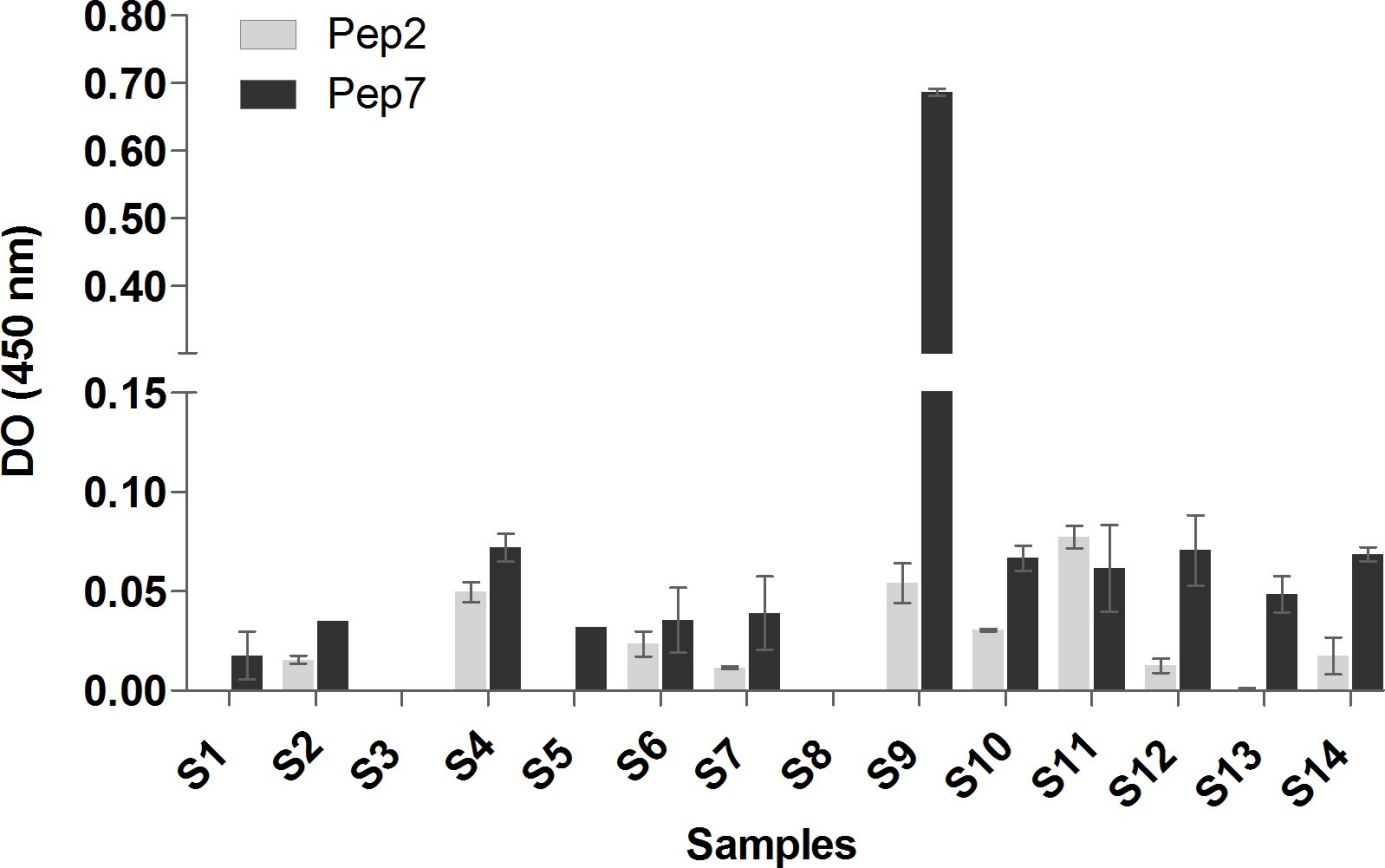
Validation of Pep2 and Pep7 of LruC protein by recognition of antibodies from sera of leptospirosis patients, by ELISA. The ELISA measured the recognition of the selected peptides by the sera of leptospirosis patients (S1 to S14). The graphic represents the average and standard deviation of optic density (DO) of the duplicates using 10μg of peptides incubated with the human sera. The values were subtracted by the background recognition of control serum.

Validation of the peptides Pep2 and Pep7 by ELISA showed highest recognition of the Pep7 when compared to Pep2 (Fig 5 and S6 Table in S1 File). The results were reproductive using the different peptide concentrations, including the very high recognition of Pep7 by serum S9. The recognition did not correlate directly with MAT titles (S6 Table in S1 File).

### Expression of LruC gene in organs of infected mice

**Fig 6:**
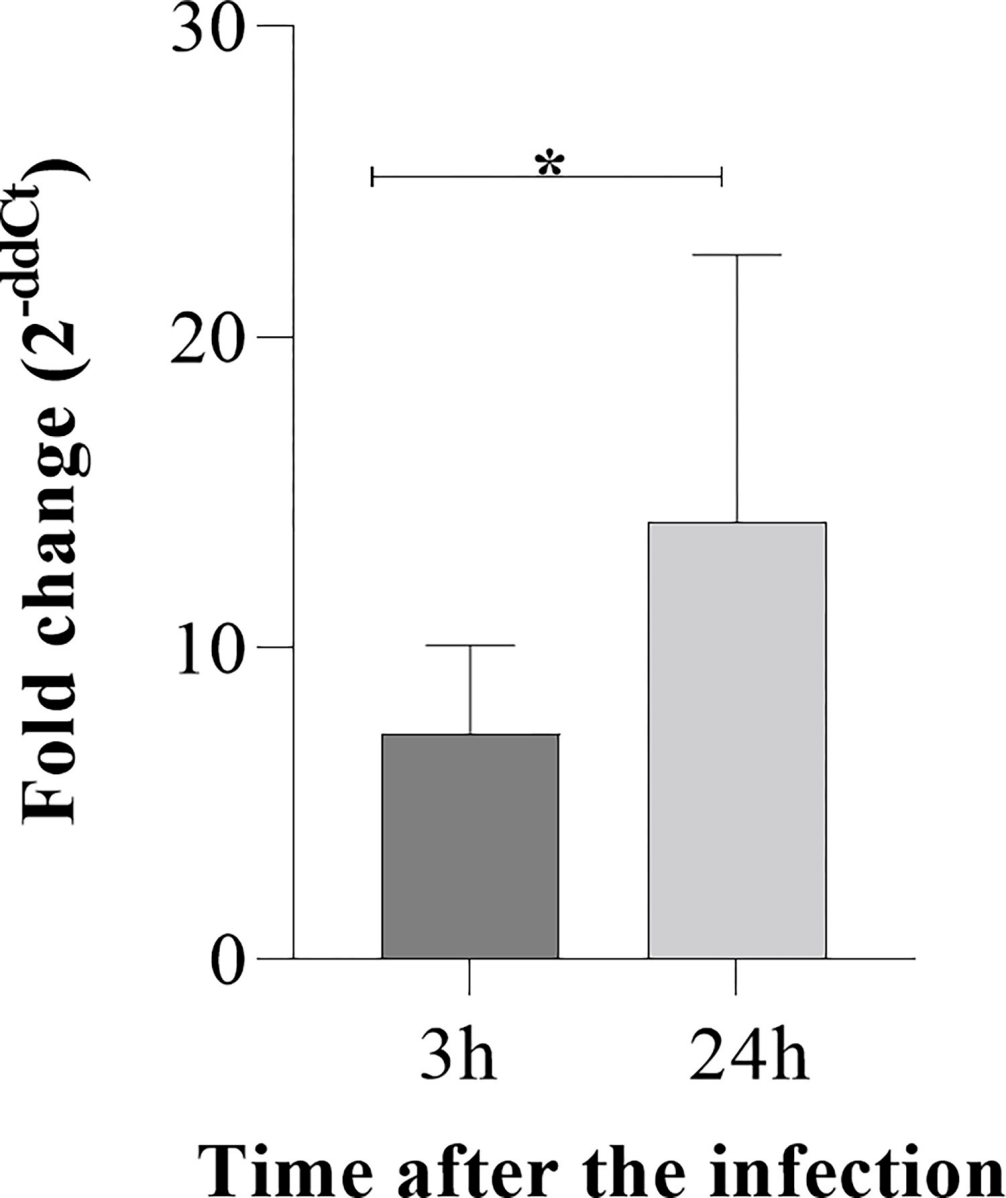
LruC expression in lung of mice. C3H/HeJ mice (n = 6) were infected with 10^*7*^ *L*. *interrogans* serovar Copenhageni. Statistical differences analysis were performed using Prism software (GraphPad). *p<0.05.

*Leptospira* LruC expression in organs of infected mice was assessed through the quantification of mRNA levels in susceptible animal model C3H/HeJ mice, by qPCR. Samples were obtained as described previously [19]. Lungs, liver and kidneys were collected 3 h and 24 h after the infection. We observed higher levels of LruC in lungs after 24 h when compared to 3 h (Fig 6), while no statistical difference of LruC expression in liver and kidneys was found. The increased of expression of LruC in lungs of animals infected with *Leptospira* suggests a role in pathogenesis in the lungs, thus confirming that the derived peptides are interesting to continue the studies for their use on vaccines and diagnoses.

## Discussion

Peptide-based vaccines can be formulated with conserved epitopes froma protein of different serotypes and or even by multiple non-contiguous immunodominant epitopes from pathogens in order to culminate in broad-spectrum vaccines [4, 22]. Currently, the reverse vaccinology (RV) approach has been used to identify potential proteins and peptides that fit the immunodominant requirement that may display the best features to provide cross-immunity against *Leptospira* serovars [22–25].

RV has been applied for different pathogens, such as *Pasteurella multocida*, *Influenza*, and *Hepatitis* C [22, 26, 27] as well as for cancer treatments, haematology, cardiovascular and autoimmune diseases [28]. A few studies tried to accomplish the same strategy for *Leptospira* [6]. This investigation was conducted to analyze LruC immune prevalence during *Leptospira* pathogenesis and to identify epitopes that are able to trigger the adaptive immune response, thus recognized by antibodies, what would be crucial for host protection.

LruC is an inner membrane protein, highly conserved among most *Leptospira* pathogenic species. Therefore, it has been suggested as an interesting vaccine candidate by immunoinformatics due to its localization, conservation and immunogenicity [17]. Immunoinformatics tools and peptide design were used to analyze LruC immune features, resulting that the protein has several conserved and immunogenic epitopes, which could be explored for development of a broad-spectrum vaccine. Lata and collaborators (2018) pointed out a few *Leptospira* peptides that could be used for vaccine investigation [16, 17]. Six of them were related to LruC protein.

Our *in silico* analysis showed that some of these peptides are presented to MHC complex I and II (Table 1 and S3 Table in S1 File), which is important for antigen presentation. Peptide-HLA prediction tools also helped to filter relevant epitopes for vaccine development. This strategy had been used for different diseases, such as *Mycobacterium tuberculosis* and cancer [29, 30]. For instance, Kanagavel and collaborators (2014) [31], have used prediction tools to design *Leptospira* LigA epitopes to be used in diagnose by ELISA and it reached almost 6% more sensitivity than the usual ELISA antigens [31]. Our results of peptide validation by ELISA also confirm that this strategy is efficient to be used to choose peptides for development of vaccine and diagnose. The analysis by NetMHC 4.0 on the protein LruC indicates a series of overlapping peptides that show strong bind to MHC I and II, which include Pep7, a 8 amino acid sequence published by Lata and collaborators [16]. Pep7 binds to HLA, according NetMHC, and this epitope is conserved in various *Leptospira* species, as showed by BLASTp.

After *in silico* analysis, all seven peptides were tested by Dot Blot assay using the sera of hamsters immunized with low-LPS bacterin. Pep1, Pep2 and Pep7 seemed to be the most promising, as they were recognized only by antibody from immunized hamsters (Fig 3), implying that these epitopes trigger the adaptive response during immunization, resulting in antibodies production. Our results indicate that the strategy is interesting and could be further explored.

We tested Pep2 and Pep7 against the serum of patients diagnosed with leptospirosis. Pep2 presented low signal in dot assay and the control sera also displayed a signal. Pep7 was strongly detected, showing good specificity, in agreement with our *in silico* analysis, which indicated that Pep7 has features of strong binding to MHC.

Few other *Leptospira* proteins have been used as a source of potential peptides for vaccine development. For example, epitopes from OmpL1 and LipL41 were able to induce lymphocyte differentiation and antibody production, as well as epitopes from LmpL1, LipL32, and LipL21 fused as one molecule [32, 33]. The identification of new proteins and epitopes such as Pep7 increases the likelihood of success in developing peptide-based vaccine or diagnostic.

Pep4, it exhibited interaction with both sera from immunized and control animals, evidencing nonspecific interaction with antibodies. This could be related to the fact that Pep4 present match with several proteins by BLASTp, and its analysis by HPLC exhibited certain impurities suggesting possible interference on the assay (S1 Fig).

Pep3, Pep5, and Pep6, although considered having good MHC binding properties, they did not display significant difference on the interaction with antibodies of sera from immunized or control hamsters in Dot Blot assay. Although these peptides are considered having good MHC binding properties, they were not recognized in the Dot Blot assay.

Our results showed that LruC is up-regulated in lung of C3H/HeJ mice infected with *L interrogans* serovar Copenhageni, suggesting a role of the protein in virulence during the infection, such as other leptospire proteins [34]. Likewise, other bacteria can trigger and modulate virulence factors expression when it is necessary. For example, *Helicobacter pylori*, bacteria responsible for gastric disorders, is able to regulate its OMPs and virulence factors to adapt itself to the environment and *Staphylococcus aureus* sense iron alterations and alters its virulence factors profile [35, 36]. Different from the lung, there was no statistical significances in LruC expression in the kidneys and liver. This could suggest a modulation on *Leptospira* transcriptome at different environment and conditions during infection, but further experiments need to be better investigate.

Our results indicating that LruC is expressed during infection and might be acting as a virulence factor reinforces its potential for prospective studies on vaccines and diagnostic methods. In this context we identified a promising peptide, conserved and specifically recognized by the antibodies from bacterin immunized animals and from leptospirosis patients. It could increase the chance on the search for a broad-spectrum protection vaccine.

## Conclusion

Immunoinformatics analysis was an important tool used in this study to identify peptides that could interact with the MHC complex. Among the seven selected peptides, Pep7 was better recognized by antibodies from leptospirosis patients in our studies and it was validate by ELISA. Pep7 stood out for further investigation in vaccines or diagnostic studies.

## Supporting information

S1 Fig HPLC chromatogram representing the peak of selected peptide after purification. (TIF)

S2 Fig Original membranes from dot blot assay. (PDF)

S1 File (XLS)

## References

[1] Asoh T, Saito M, Villanueva SY, Kanemaru T, Gloriani N, Yoshida S. Natural defense by saliva and mucosa against oral infection by Leptospira. Can J Microbiol. 2014;60(6):383–9. doi: 10.1139/cjm-2014-0016 24861456

[2] Grassmann AA, Souza JD, McBride AJ. A Universal Vaccine against Leptospirosis: Are We Going in the Right Direction? Front Immunol. 2017;8:256. doi: 10.3389/fimmu.2017.00256 28337203 PMC5343615

[3] Costa F, Hagan JE, Calcagno J, Kane M, Torgerson P, Martinez-Silveira MS, et al. Global Morbidity and Mortality of Leptospirosis: A Systematic Review. PLoS Negl Trop Dis. 2015;9(9):e0003898. doi: 10.1371/journal.pntd.0003898 26379143 PMC4574773

[4] Cosson JF, Picardeau M, Mielcarek M, Tatard C, Chaval Y, Suputtamongkol Y, et al. Epidemiology of leptospira transmitted by rodents in southeast Asia. PLoS Negl Trop Dis. 2014;8(6):e2902. doi: 10.1371/journal.pntd.0002902 24901706 PMC4046967

[5] Teixeira AF, Fernandes LGV, Cavenague MF, Takahashi MB, Santos JC, Passalia FJ, et al. Adjuvanted leptospiral vaccines: Challenges and future development of new leptospirosis vaccines. Vaccine. 2019;37(30):3961–73. doi: 10.1016/j.vaccine.2019.05.087 31186193

[6] Adler B. (2015). Vaccines Against Leptospirosis. In: Adler B. (eds) Leptospira and Leptospirosis. Current Topics in Microbiology and Immunology, vol 387. Springer, Berlin, Heidelberg. 10.1007/978-3-662-45059-8_10. 25388138

[7] Haake DA, Levett PN. Leptospirosis in humans. Curr Top Microbiol Immunol. 2015;387:65–97. doi: 10.1007/978-3-662-45059-8_5 25388133 PMC4442676

[8] Li W, Joshi MD, Singhania S, Ramsey KH, Murthy AK. Peptide Vaccine: Progress and Challenges. Vaccines (Basel). 2014;2(3):515–36. doi: 10.3390/vaccines2030515 26344743 PMC4494216

[9] Lucas DS, Cullen PA, Lo M, Srikram A, Sermswan RW, Adler B. Recombinant LipL32 and LigA from Leptospira are unable to stimulate protective immunity against leptospirosis in the hamster model. Vaccine. 2011;29(18):3413–8. doi: 10.1016/j.vaccine.2011.02.084 21396409

[10] Felix CR, Siedler BS, Barbosa LN, Timm GR, McFadden J, McBride AJA. An overview of human leptospirosis vaccine design and future perspectives. Expert Opin Drug Discov. 2020;15(2):179–88. doi: 10.1080/17460441.2020.1694508 31777290

[11] Dellagostin OA, Grassmann AA, Hartwig DD, Félix SR, da Silva É, McBride AJ. Recombinant vaccines against leptospirosis. Hum Vaccin. 2011;7(11):1215–24. doi: 10.4161/hv.7.11.17944 22048111

[12] Yang X, Yu X. An introduction to epitope prediction methods and software. Rev Med Virol. 2009;19(2):77–96. doi: 10.1002/rmv.602 19101924

[13] Patronov A, Doytchinova I. T-cell epitope vaccine design by immunoinformatics. Open Biol. 2013;3(1):120139. doi: 10.1098/rsob.120139 23303307 PMC3603454

[14] Larsen JE, Lund O, Nielsen M. Improved method for predicting linear B-cell epitopes. Immunome Res. 2006;2:2. doi: 10.1186/1745-7580-2-2 16635264 PMC1479323

[15] Dellagostin OA, Grassmann AA, Rizzi C, Schuch RA, Jorge S, Oliveira TL, et al. Reverse Vaccinology: An Approach for Identifying Leptospiral Vaccine Candidates. Int J Mol Sci. 2017;18(1). doi: 10.3390/ijms18010158 28098813 PMC5297791

[16] Lata KS, Kumar S, Vaghasia V, Sharma P, Bhairappanvar SB, Soni S, et al. Exploring Leptospiral proteomes to identify potential candidates for vaccine design against Leptospirosis using an immunoinformatics approach. Sci Rep. 2018;8(1):6935. doi: 10.1038/s41598-018-25281-3 29720698 PMC5932004

[17] Verma A, Matsunaga J, Artiushin S, Pinne M, Houwers DJ, Haake DA, et al. Antibodies to a novel leptospiral protein, LruC, in the eye fluids and sera of horses with Leptospira-associated uveitis. Clin Vaccine Immunol. 2012;19(3):452–6. doi: 10.1128/CVI.05524-11 22237897 PMC3294619

[18] Lauretti-Ferreira F, Silva PLD, Alcântara NM, Silva BF, Grabher I, Souza GO, et al. New strategies for Leptospira vaccine development based on LPS removal. PLoS One. 2020;15(3):e0230460. doi: 10.1371/journal.pone.0230460 32218590 PMC7100938

[19] Silva PL, Nakajima E, Costa RMD, Lee Ho P, Martins EA, Carvalho E, et al. Chemokine expression profiles in liver and kidney of mice with different susceptibilities to leptospirosis. Microb Pathog. 2020;149:104580. doi: 10.1016/j.micpath.2020.104580 33080359

[20] Reynisson B, Barra C, Kaabinejadian S, Hildebrand WH, Peters B, Nielsen M. Improved Prediction of MHC II Antigen Presentation through Integration and Motif Deconvolution of Mass Spectrometry MHC Eluted Ligand Data. J Proteome Res. 2020;19(6):2304–15. doi: 10.1021/acs.jproteome.9b00874 32308001

[21] Nielsen M, Lundegaard C, Worning P, Lauemøller SL, Lamberth K, Buus S, et al. Reliable prediction of T-cell epitopes using neural networks with novel sequence representations. Protein Sci. 2003;12(5):1007–17. doi: 10.1110/ps.0239403 12717023 PMC2323871

[22] Molero-Abraham M, Lafuente EM, Flower DR, Reche PA. Selection of conserved epitopes from hepatitis C virus for pan-populational stimulation of T-cell responses. Clin Dev Immunol. 2013;2013:601943. doi: 10.1155/2013/601943 24348677 PMC3856138

[23] Oli AN, Obialor WO, Ifeanyichukwu MO, Odimegwu DC, Okoyeh JN, Emechebe GO, et al. Immunoinformatics and Vaccine Development: An Overview. Immunotargets Ther. 2020;9:13–30. doi: 10.2147/ITT.S241064 32161726 PMC7049754

[24] Dalsass M, Brozzi A, Medini D, Rappuoli R. Comparison of Open-Source Reverse Vaccinology Programs for Bacterial Vaccine Antigen Discovery. Front Immunol. 2019;10:113. doi: 10.3389/fimmu.2019.00113 30837982 PMC6382693

[25] Baseer S, Ahmad S, Ranaghan KE, Azam SS. Towards a peptide-based vaccine against Shigella sonnei: A subtractive reverse vaccinology based approach. Biologicals. 2017;50:87–99. doi: 10.1016/j.biologicals.2017.08.004 28826780

[26] Tatum FM, Tabatabai LB, Briggs RE. Cross-protection against fowl cholera disease with the use of recombinant Pasteurella multocida FHAB2 peptides vaccine. Avian Dis. 2012;56(3):589–91. doi: 10.1637/9991-111611-ResNote.1 23050479

[27] Lee JS, Chowdhury MY, Moon HJ, Choi YK, Talactac MR, Kim JH, et al. The highly conserved HA2 protein of the influenza A virus induces a cross protective immune response. J Virol Methods. 2013;194(1–2):280–8. doi: 10.1016/j.jviromet.2013.08.022 24004822

[28] Trier N, Hansen P, Houen G. Peptides, Antibodies, Peptide Antibodies and More. Int J Mol Sci. 2019;20(24). doi: 10.3390/ijms20246289 31847088 PMC6941022

[29] Teku GN, Vihinen M. Pan-cancer analysis of neoepitopes. Sci Rep. 2018;8(1):12735. doi: 10.1038/s41598-018-30724-y 30143704 PMC6109115

[30] Khanna D, Rana PS. Ensemble Technique for Prediction of T-cell Mycobacterium tuberculosis Epitopes. Interdiscip Sci. 2019;11(4):611–27. doi: 10.1007/s12539-018-0309-0 30406342

[31] Kanagavel M, Shanmughapriya S, Anbarasu K, Natarajaseenivasan K. B-cell-specific peptides of leptospira interrogans LigA for diagnosis of patients with acute leptospirosis. Clin Vaccine Immunol. 2014;21(3):354–9. doi: 10.1128/CVI.00456-13 24403522 PMC3957670

[32] Lin X, Xiao G, Luo D, Kong L, Chen X, Sun D, et al. Chimeric epitope vaccine against Leptospira interrogans infection and induced specific immunity in guinea pigs. BMC Microbiol. 2016;16(1):241. doi: 10.1186/s12866-016-0852-y 27737644 PMC5064800

[33] Lin X, Sun A, Ruan P, Zhang Z, Yan J. Characterization of conserved combined T and B cell epitopes in Leptospira interrogans major outer membrane proteins OmpL1 and LipL41. BMC Microbiol. 2011;11(1):21. doi: 10.1186/1471-2180-11-21 21269437 PMC3038132

[34] Hsu SH, Yang CW. Insight into the Structure, Functions, and Dynamics of the. Membranes (Basel). 2022;12(3). 10.3390/membranes12030300 PMC8951592 35323775

[35] Torres VJ, Attia AS, Mason WJ, Hood MI, Corbin BD, Beasley FC, et al. Staphylococcus aureus fur regulates the expression of virulence factors that contribute to the pathogenesis of pneumonia. Infect Immun. 2010;78(4):1618–28. doi: 10.1128/IAI.01423-09 20100857 PMC2849423

[36] Xu C, Soyfoo DM, Wu Y, Xu S. Virulence of Helicobacter pylori outer membrane proteins: an updated review. Eur J Clin Microbiol Infect Dis. 2020;39(10):1821–30. doi: 10.1007/s10096-020-03948-y 32557327 PMC7299134

