# Supplementary figures and images for: Analysis of LruC lipoprotein and identification of peptides candidates for vaccine development and diagnosis of leptospirosis
Source: PLoS One. 2023 Feb 6;18(2):e0281344. doi: 10.1371/journal.pone.0281344 (PMC9901810; doi:10.1371/journal.pone.0281344)

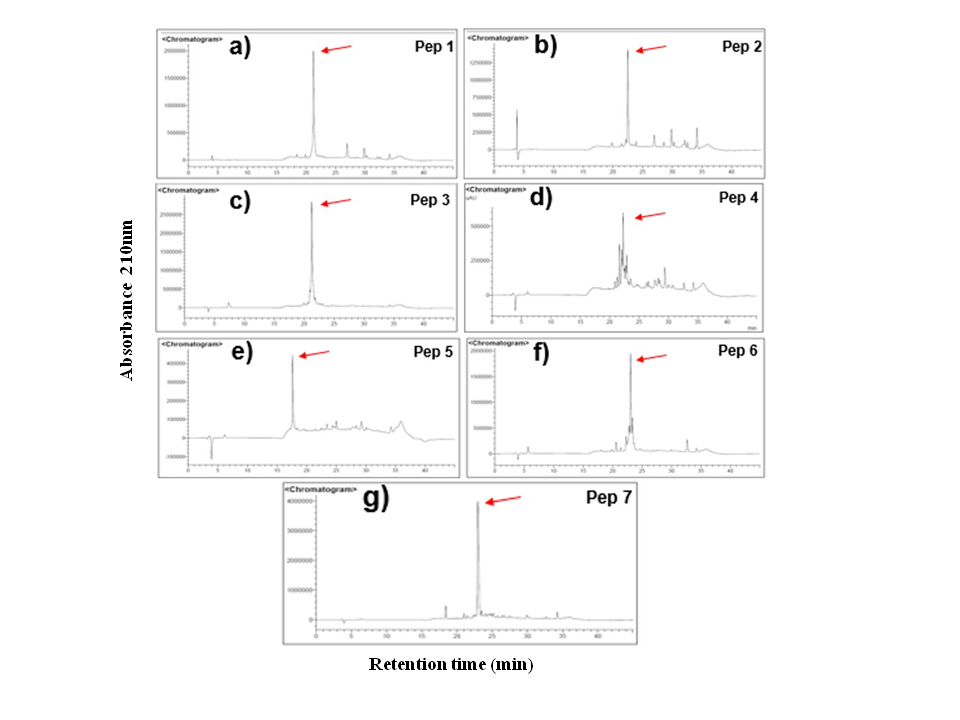

Supplement: S1 Fig — (TIF) [file pone.0281344.s001.tif]
