# Supplementary material for: Analysis of LruC lipoprotein and identification of peptides candidates for vaccine development and diagnosis of leptospirosis
Source: PLoS One. 2023 Feb 6;18(2):e0281344. doi: 10.1371/journal.pone.0281344 (PMC9901810; doi:10.1371/journal.pone.0281344)

Supporting Information

Analysis of LruC lipoprotein and identification of peptides candidates for vaccine development and diagnosis of leptospirosis

Iago H. de Miranda Mariano[1,], Bruna Ferreira Silva[1,2], Mayriele da S Machado[1,], Roberta M Blanco[3], Eliete C Romero[3], Sonia A Andrade[4], Paulo Lee Ho[5], Elizabeth AL Martins[6], Josefa B da Silva[1*]

[1]Laboratory of Bacteriology, Butantan Institute, Sao Paulo, Brazil.

[2]PIBITI/CNPq and Butantan Foundation.

[3]Laboratory of Bacteriology, Adolfo Lutz Institute, Sao Paulo, Brazil.

[4]Laboratory of Biopharmaceuticals, Butantan Institute, Sao Paulo, Brazil.

[5]Bioindustrial Division, Butantan Institute, São Paulo, Brazil

[6]Laboratory of Recombinant Biological, Butantan Institute, São Paulo, Brazil.

*Corresponding author: josefa.silva@butantan.gov.br (Da Silva JB).

https://orcid.org/0000-0003-3884-7111

S2 Figures - Original membranes of dot blot assays utilized to set figures 3 (hamster sera)

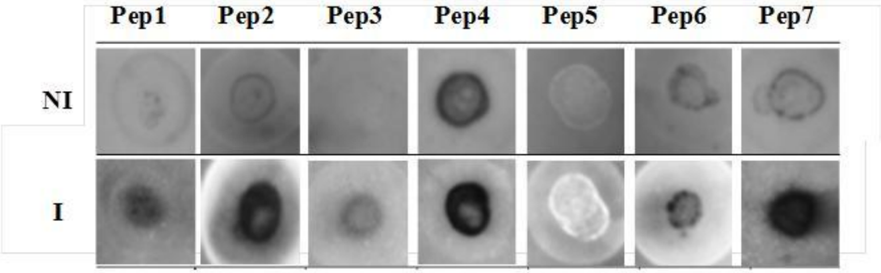

Fig 3: Recognition of peptides by antibodies in the sera of immunized hamsters.

Original membranes of dot blot assay shown in figure 3

NI

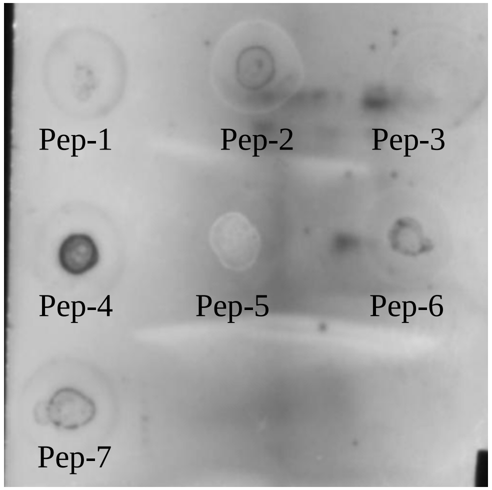

Control: Dot Blot assay using serum of no immunized hamster (NI)

I

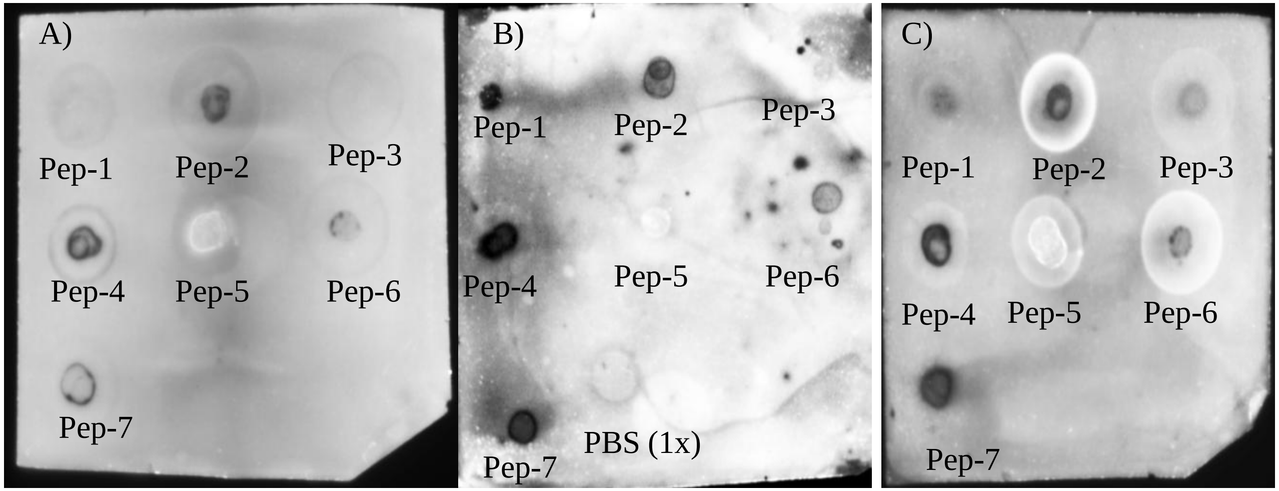

Dot Blot assay using serum of immunized hamster (I). A) Vaccine serovar Canicola, B) Vaccine serovars Canicola and Copenhageni (second bleeding), C) Vaccine serovars Canicola and Copenhageni and challenged group.

Original membranes of dot blot assays utilized to set figures 4 (S1 – S4 human sera)

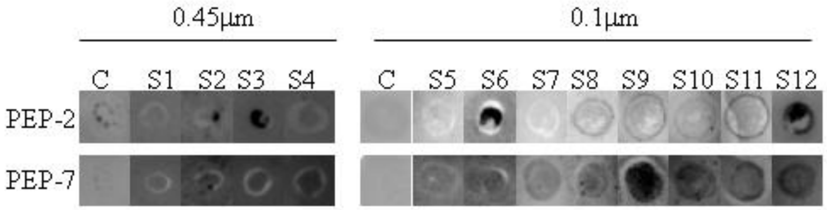

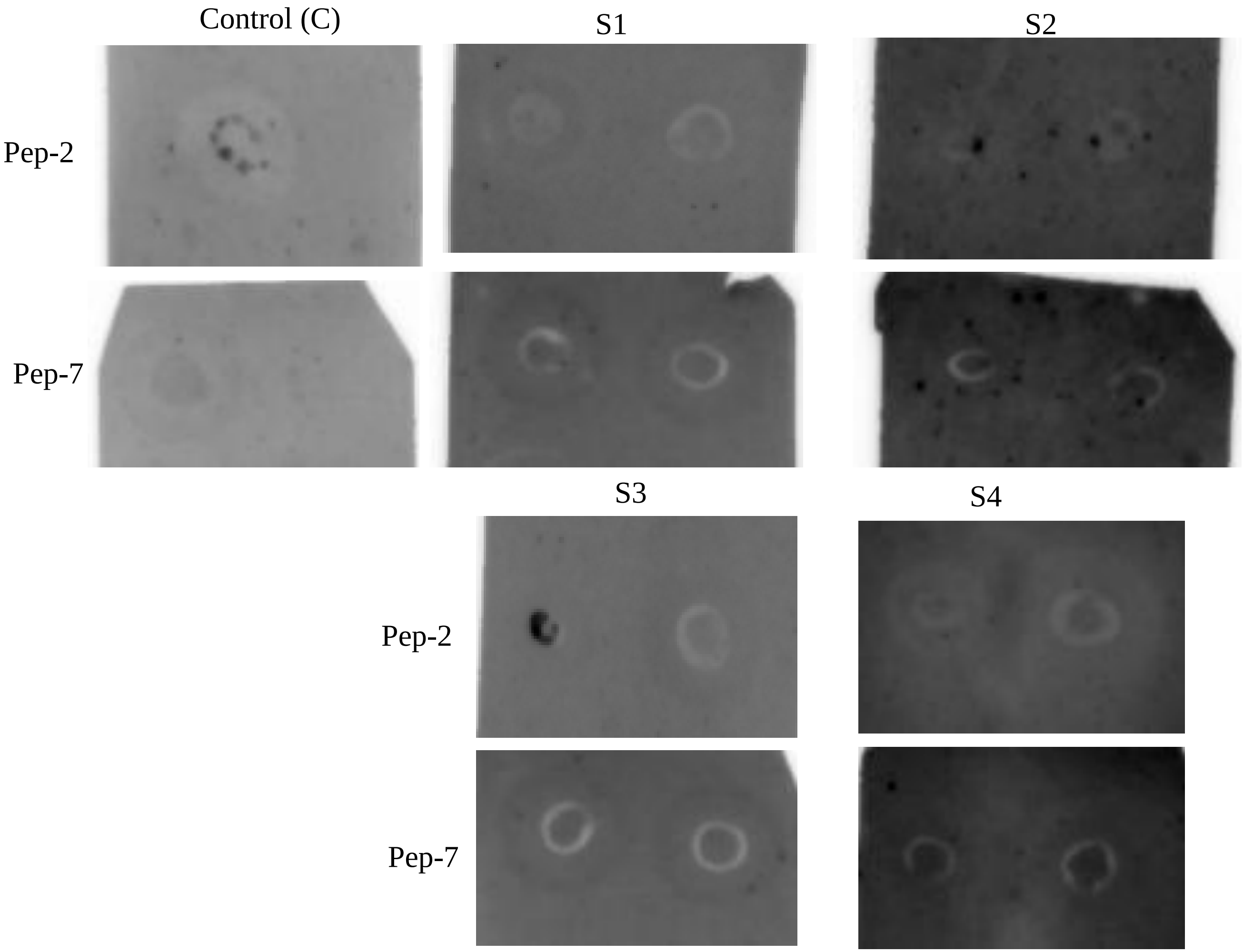

Original membranes of dot blot assays utilized to set figures 4 (S5 – S12 human sera)

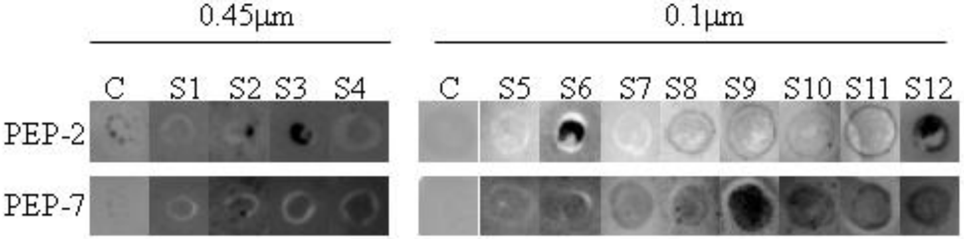

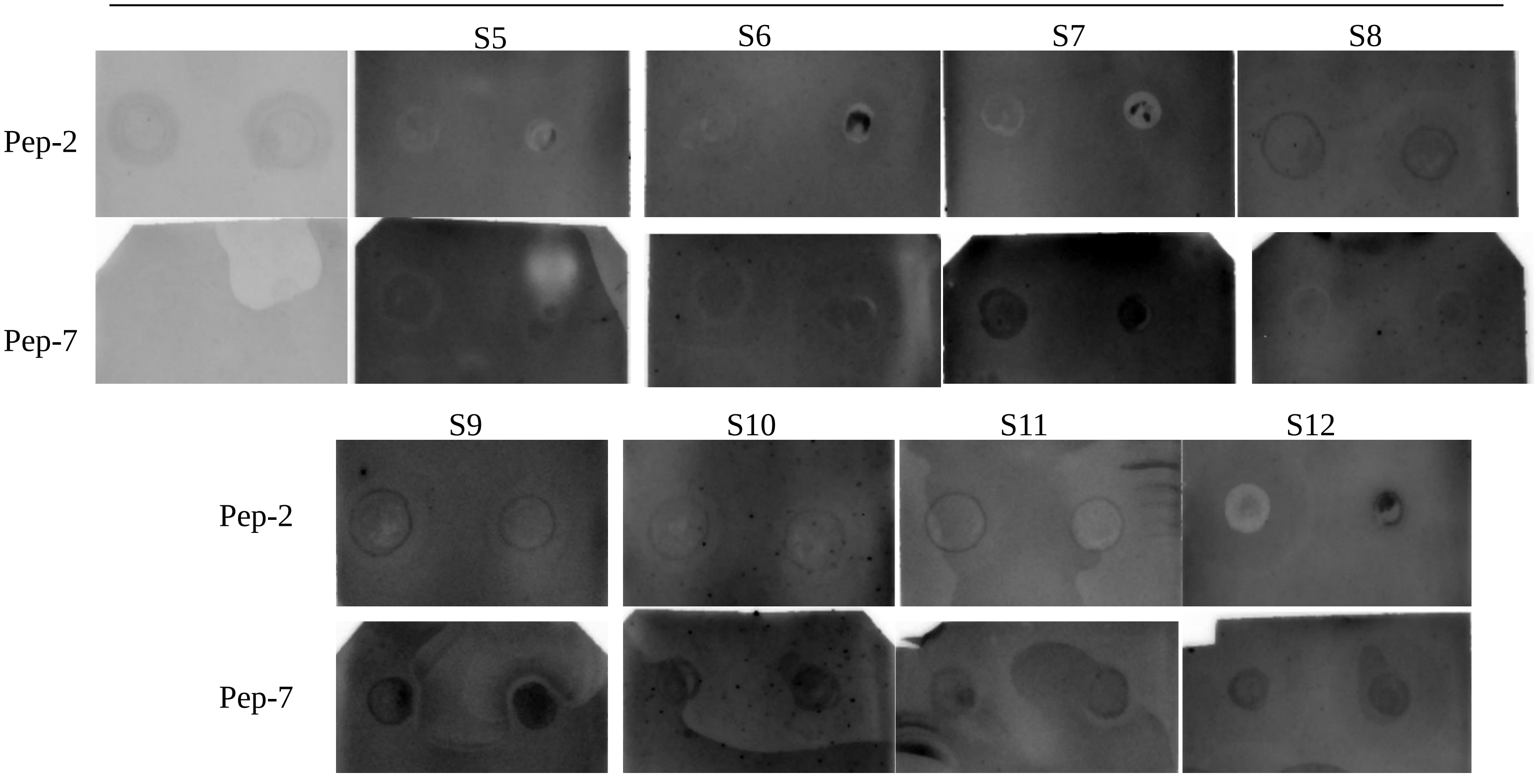

Supplement: S2 Fig — (PDF) [file pone.0281344.s002.pdf]
